# The newborn behavioural observations (NBO) system embedded in routine postpartum care in at-risk families in Iceland: a randomised controlled trial

**DOI:** 10.1186/s12884-024-07128-0

**Published:** 2025-01-08

**Authors:** Lisbeth Valla, Kari Slinning, Tore Wentzel-Larsen, Gun-Mette Røsand, Stefanía Birna Arnardóttir

**Affiliations:** 1 Regional Centre for Child and Adolescent Mental Health, Eastern and Southern Norway (RBUP), Oslo, Norway; 2 Department of Nursing and Health Promotion, Faculty of Health Sciences, Oslo Metropolitan University, Oslo, Norway; 3 Psychiatric team for prospecting parents and parents with young children, Primary health care in capital area, Reykjavik, Iceland; 4 Norwegian Centre for Violence and Traumatic Stress Studies, Oslo, Norway

**Keywords:** Newborn behaviour observation, NBO, Mother-infant interaction

## Abstract

**Background:**

The Newborn Behaviour Observation system (NBO) is a flexible relationship-based intervention designed to sensitise parents to their newborn’s capacities, to increase parental confidence and foster the bond between parent and infant. The aim of this study was to investigate the effects of an NBO intervention on maternal confidence during the first month postpartum, and on the quality of mother-infant interaction at infant age 4 months in a sample of mothers who exhibit elevated signs of distress or depression during pregnancy and/or describe prior experiences of mental health issues.

**Method:**

Pregnant women with current emotional distress and/or a history of anxiety and depression were recruited from a healthcare centre in Reykjavik, between August 2016 and April 2018. The study used a two-group, randomised trial design with six measuring points, in which 54 women were randomly assigned to either the intervention or control group. The intervention group (*n* = 26) received the NBO in combination with standard care during routine home visits. The control group (*n* = 28) received the same numbers of home visits with standard care without NBO. Maternal confidence was measured using a parent questionnaire (covering learning outcomes relating to the infant’s communicative signals and maternal confidence) administered after each home visit in weeks 2, 3 and 4 postpartum. At 4 months infant age, a free-play situation involving mother-infant interaction was video-recorded in the participants’ homes and coded using the Emotional Availability Scale (EAS). Mixed effects models were used to estimate group differences in learning outcomes and maternal confidence across three time points. Two sample t-tests were used to compare the two groups’ EAS scores.

**Results:**

The mothers in the intervention-group reported significantly higher maternal confidence and increased knowledge about their infant compared to the control group. Adjusted analyses suggest some evidence of a higher EAS non-hostility score in the intervention group (*p* = .031), but not for the other EAS scale scores (*p* ≥ .118).

**Conclusion:**

Early home visits combining NBO with standard care enhance maternal confidence and the mother’s understanding of her infant. The small sample size makes it difficult to conclude whether repeated NBO sessions during the first month increase dimensions of maternal sensitivity in mother-infant interaction at 4 months postpartum.

**Trial registration:**

ClinicalTrials.gov ID NCT04739332, Registered 02/01/2021.

## Introduction

The quality of the early relationship between a primary caregiver and child during infancy exerts an especially strong influence on child development [1]. It is acknowledged that sensitive and responsive caregiving is associated with positive infant development in several areas, including socio-emotional, cognitive and behavioural development [2, 3]. Positive parent-infant interactions depend on the parents’ ability to respond sensitively to the child’s individually expressed signals [4], and their understanding and attunement to the infant’s internal state and behaviour [5]. In most parents, these caregiving behavioural and regulation skills evolve intuitively during pregnancy and after birth [6]. However, it has been shown that maternal distress and mental illness in the perinatal period may disturb the development of sensitive and attuned emotional parent-infant interaction during early infancy [7, 8]. Depressed mothers show increased latencies in repairing interactive mismatches into positive- matched states [9], and parents with depression, anxiety or other life stressors tend towards a more negative (hostile and intrusive) and less responsive parenting style [10, 11]. Furthermore, they may touch and talk less with their infant and may show more negative facial expressions during face-to-face interaction [12], which may negatively impact the social interaction between the infant and the primary caregiver and impact the mother-infant dyad.

Previous research provides overall support for preventive interventions that directly target maternal postpartum depression [13], but interventions that solely target the mother’s symptoms do not guarantee a sensitive and responsive mother-infant interaction pattern or positive child outcomes [14, 15]. When the goal is to reduce the risk of negative effects on the offspring, interventions that target the caregiver-infant interaction are needed [16]. The Newborn Behaviour Observation system (NBO) is a brief, relationship-based intervention designed to sensitise parents to their newborn’s capacities and individuality, with the goal of fostering the bond between parent and infant [17]. Specifically, the NBO involves a set of shared observations of the newborn infant together with the parent. NBO includes a series of manoeuvres that are used to [1] elicit infant neurobehaviors, and [2] describe and interpret these neurobehaviors in the context of the parent-infant interaction and infant self-regulation and intentions. This interactive approach is designed to promote mentalisation or parental reflective functioning, by enabling the parent to think about and understand her child’s feelings and experiences, and see the infant as an individual, thus enhancing the parent–infant relationship.

Studies of NBO have shown positive outcomes in the form of higher maternal confidence in important everyday situations with the infant [18–20]. However, only a few studies have investigated effects of NBO on observed mother-child interactions [21]. A systematic review of the Newborn Behaviour Assessment Scale (NBAS) and NBO from 2018 found only three NBO studies that satisfied the inclusion criteria. The review concluded that existing results are inconclusive because of small sample sizes with mainly low-risk, first-time mothers [22]. A more recently published Australian NBO study was conducted with at-risk mothers and found that the NBO intervention both improved mother-infant interaction at 4 months postpartum and reduced maternal distress [18]. Despite some promising research findings and great interest in the NBO among health practitioners worldwide, robust evidence of its effectiveness is key to enabling decisions about how to best support vulnerable caregivers of newborns. Since there is currently no reliable systematic review of this evidence to assist with decision-making about its use, we aim to contribute to a new review, hopefully in the near future, through this small RCT study of the effects of NBO among at-risk families in Iceland. Thus, the NBO was tested in a local healthcare centre in Reykjavik with women at risk of experiencing postpartum depression. The at-risk women who were randomly assigned to the intervention-group underwent three sessions of NBO combined with standard care delivered during regular home visits in weeks 2, 3 and 4 postpartum. The control group received the same number of home visits with standard care, but without NBO.

We hypothesised that the mothers in G1 will show higher sensitivity when interacting with their infant compared to the mothers in G2 at 4 months postpartum, the primary outcome.

For secondary outcomes, we hypothesised that the infants in G1 will show better regulatory capacity than the infants in G2 during mother-infant interaction. We also hypothesised that mothers in the intervention group (G1, NBO combined with standard care) will report higher satisfaction and benefits from the postpartum home visits compared to the mothers in the control group (G2, standard care only).

## Methods

### Design and procedure

The study was a two-group randomised trial.

Eligible participants (*n* = 56) were randomised to the intervention group (G1, *n* = 28) or the control group (G2, *n* = 28) with an allocation ratio of one-to-one. A simple random allocation procedure using a computer software programme was used to generate the random sequence. The allocation procedure was prepared by an investigator with no clinical involvement in the trial. The researchers who had the role of assessing the results were blinded. The participants were not unaware, however, of their randomisation setting. Two participants in the intervention group never completed any questionnaire. Thus, the study sample comprised 54 participants (26 intervention, 28 control). The trial ended at the original primary endpoint; 4 months postpartum.

### Recruitment and participants

**Fig. 1:**
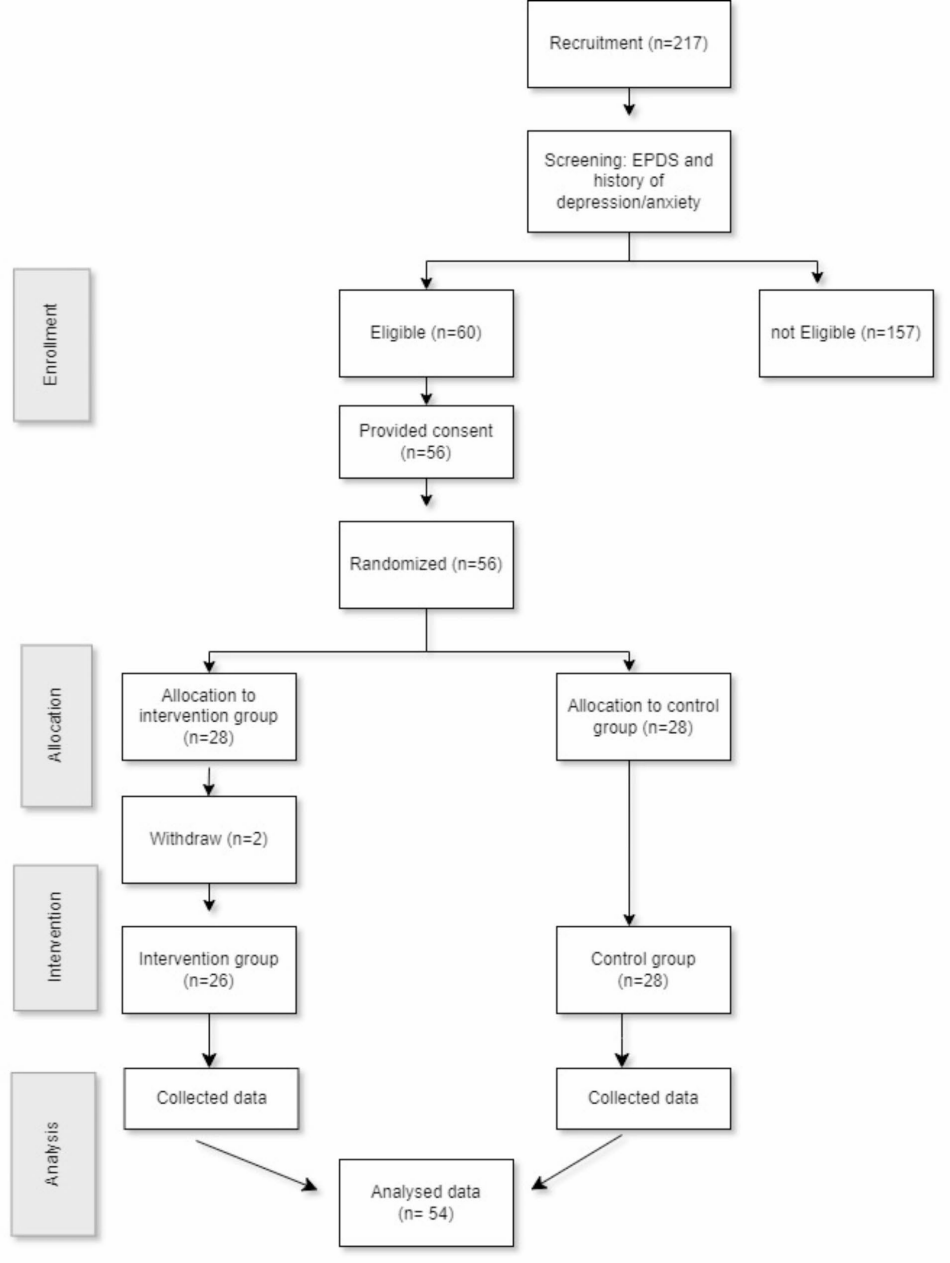
Flow diagram of the study

Participants were recruited from August 2016 to April 2018, in Reykjavik, the capital of Iceland, during routine pregnancy visits by midwives at an antenatal health care centre. As part of the routine care during the second trimester, all pregnant women are asked about their mental health and are invited to complete the Edinburgh Postnatal Depression scale (EPDS) [23]. For the current study, two additional questions were added about previous mental health problems. The study’s eligibility criteria were that the pregnant woman had at least one of the following three conditions: (1) a total score ≥ 10 on the EPDS administered in the 23rd to 28th week of pregnancy, (2) a confirmed a history of depression, (3) a history of anxiety. Additional eligibility criteria were (4) age 18 years old or above, and (5) being sufficiently proficient in spoken and written Icelandic. Women assessed for eligibility were given oral and written information about the study and invited to participate. During the recruitment period, 217 women who made up the total population completed the EPDS with the two additional questions. Among these 217, 60 women met the inclusion criteria. and 56 women accepted the invitation to participate and signed the letter of informed consent. Thus, 56 women were randomly assigned via a true randomisation process generated by a team member not involved in the study, to either the intervention or control group, and received the first questionnaire before the child was born (T1). However, two women withdrew from the study after randomisation. Participants would later be excluded if the infant was born before gestational week 35, or if the infant was born with a severe disability (none were later excluded). The study flow is showed in Fig. 1. Participants were followed until the child was 4 months old when the free-play situation of the mother-infant interaction was video-recorded. Participants did not receive any compensation for participation.

### Sample size

The sample size for this trial was determined by the number of screened pregnant women who met the inclusion criteria within a predefined recruitment period and who consented to participate in this study.

### The NBO intervention

As part of the routine home visits provided by the public health nurses in weeks 2, 3 and 4 postpartum, the G1 group received standard care in combination with the NBO. Standard care postpartum in Iceland includes daily home visits by a midwife in the first week and one home visit in weeks 2, 3 and 4 by a public health nurse. For more information, see Table 1 below.

**Table 1:** Standard care postpartum programme

Consultations Times		Content Parent guidance/ Health education/ Health examination of the child	Who	Where
Week 1	7	Maternal health and wellbeing, including parent-infant interaction and breastfeeding	Midwife	Home visit
Week 2	1	Maternal health Breastfeeding Child development and well-being, including parent-infant interaction	PHN	Home visit
Week 3	1	Maternal health Breastfeeding Child development and well-being, including parent-infant interaction	PHN	Home visit
Week 4	1	Maternal health Breastfeeding Child development and well-being, including parent-infant interaction	PHN	Home visit
Week 6	1	Health examination of the child Breastfeeding Child development and well-being, including parent-infant interaction	PHN Doctor	Healthcare Centre
Week 9	1	Maternal mental health (EPDS) Breastfeeding Child development and well-being, including parent-infant interaction	PHN	Healthcare Centre
3 months	1	Maternal health Breastfeeding Child development and well-being, including parent-infant interaction	PHN	Healthcare Centre
5 months	1	Maternal health Breastfeeding Child development and well-being, including parent-infant interaction	PHN	Healthcare Centre
Total	14			

The NBO system is a neurobehavioral observation tool designed to sensitise parents to infants’ capacities and individuality, and to enhance the parent-infant relationship by strengthening parents’ confidence and practical skills in caring for their children [17]. The intervention describes the newborn’s capacities and behavioural adaptation from birth to the third month of life, and the regulative processes triggered when facing developmental challenges. The intervention focuses on observation of the infant, combined with active involvement of the parents in a shared dialogue based on 18 neuro-behavioural items, including both observation and elicited manoeuvres to identify newborn behaviours, which are interpreted in the context of parent-infant interaction. The items include infant habituation to stimuli, infant motor development, observation of the infant’s state regulation (consciousness from deep sleeping to crying) and response to face, voice, stress and activity using the NBO system [17]. Administration of the NBO is flexible; if the infant is asleep at the beginning of the session, then the NBO begins with observation of the sleeping infant and habituation responses to light disturbances, and sound habituation. If, however, the infant is crying, then the session begins with observation of the infant’s crying pattern, intensity, state regulation and soothability [17]. The NBO observation takes around 12 to 25 min [24], and the parents are engaged as partners to strengthen their confidence and practical caring skills, thereby bringing the user perspective to the forefront. In practice, this means that topics such as infant sleep, crying, soothing, eating, nappy changing, and interacting were addressed by the health practitioner with ‘in-the-moment’ comments tailored to the infant’s shifting behavioural states during the home visits. For example, when the infant was awake and quiet, they observed the infant’s orienting responses to the mother’s voice and face, and how the infants signalled a need for breaks. When the infant cried, effective soothing techniques were explored together with the mother, and when the infant was breastfeeding, they observed and explored how effectively the infant sucked. All home visits in the intervention group in weeks 2, 3 and 4 were carried out by the only public health nurse who was certified for the NBO system.

At the end of the NBO encounter, the provider and parent reflected on the shared observations and jointly developed strategies to assist the infant in self-regulation and to promote the parent–infant relationship.

### Standard care

Participants in G2 received standard care postpartum based on the regular follow-up programme for mothers and children used by Icelandic health care centres. Standard care includes reviews and follow-up after birth as required (see Table 1).

The public health nurses who delivered standard care to the participants in the control group (G2) during home visits were not familiar with the NBO system. Thus, they discussed issues with the mothers related to their birth experiences, the mother’s health, nursing, and parent-infant interaction, and they observed the infant if she/he was awake. In general, standard care often involves a form of guidance delivered as part of a conversation with the parents, while the infant is sleeping or breastfeeding.

### Data collection

Data were collected at five time points after the eligible women consented to participate. See Table 2 for an overview. Data were mainly gathered using self-administered online questionnaires in addition to videorecording of interaction in mother-infant dyads. Eligible women filled in the demographic questionnaire pre-birth (information about household income, parity, educational level and the number of children living in the household (T1)). After each home visit in weeks 2, 3 and 4 postpartum (3 times in total), the mothers in both groups completed an electronic version of the parent questionnaire (T2, T3 and T4). A 20-minute free-play situation of mother-infant interaction was video-recorded in the participants’ homes 4 months postpartum (T5). All video-recordings were conducted by the public health nurse who had made the previous home visits.

**Table 2:** Data collection schedule

Methods	Target	T0	T1	T2	T3	T4	T5
EPDS and 2 questions about history of depression and anxiety	All participants (G1, G2)	x					
Demographics Questionnaire	All participants (G1, G2)		**x**				
The Parent questionnaire	All participants (G1, G2)			x	x	**x**	
Videorecording of mother-infant free-play interaction and coding using the Emotional availability scale	Mother-infant dyads, all participants (G1, G2)						x

T0 = Recruitment 24–30 gestational week, T1 = Prenatal- ≥28 gestational week, T2 = 2 weeks postpartum, T3 = 3 weeks postpartum, T4 = 4 weeks postpartum, T5 = 4 months postpartum

### Outcomes

The primary outcome measure was the quality of mother-infant interaction (mothers’ sensitivity when interacting with their infant and infants’ regulatory capacity), specifically measured at 4 months postpartum. This was assessed through a video-recorded free-play session between mother and infant, which was later coded using the Emotional Availability Scales (EAS).

A secondary outcome measure was the mothers’ satisfaction and benefits from the postpartum home visits. This was assessed using specific items from the parent questionnaire related to competence (parents’ competence and child competence). The results were reported after each home visit in weeks 2, 3, and 4 postpartum.

#### Measures

##### EPDS – the Edinburgh postnatal depression scale (T0)

At baseline (T0), maternal depressive symptoms were measured using the Icelandic version of the Edinburgh Postnatal Depression Scale (EPDS) [23] upon recruitment. The EPDS is a ten-item, self-report screening measure used to assess depressive symptoms in the perinatal period. Symptoms are rated on a 4-point scale (0–3), with higher scores indicating increased symptom severity. Total scores were dichotomised using a cut-off of ≥ 10 to indicate potential depression. A validation study for the Icelandic version of the EPDS is not yet available. Two tests of reliability have confirmed the homogeneity of the translated Icelandic version in a national sample of 734 postpartum mothers (Cronbach’s alpha 0.87; split-half 0.83) [25, 26]. Cronbach’s alpha for women in this study was 0.81 pre‐test and 0.89 post‐test.

##### A history of depression or anxiety (T0)

When completing the EPDS, the participants were asked to respond to two additional questions by answering ‘yes’ or ‘no’: ‘Have you suffered from depression in the past?’ and ‘Have you suffered from anxiety that disrupted your daily life in the past?’

At T1, the prospective mothers completed an online demographic questionnaire including questions about the mother’s age, educational level, work position, household income, civil status, and the number of children living in the household. The newborn’s gestational age and birth weight were filled in by a research assistant after birth.

##### The parent questionnaire (T2-T4)

The parent questionnaire [17] was originally a questionnaire about parents’ experience of an NBO session. The instrument consists of a set of statements divided into four subscales /covering four areas: whether the parent has acquired new knowledge about the infant’s competence, how the parent can respond and interact with the infant, whether the observation session contributed to positive feelings towards the infant, whether the observation contributed to a positive alliance between the parent and clinician. In the current study, we used 7 items from the questionnaire in addition to one self-composed question about infant sleep patterns (8 items in total). Four items covered how much the parent learned about the child’s competence, which we decided to call ‘the Child competence scale’, and four questions were about parent competence, called ‘the Parent competence scale’, see the questions under Table 3 below. Responses were on a 4-point Likert scale (1 = nothing to 4 = a lot). All references to the NBO were excluded and both groups completed the questionnaire.

**Table 3:** Group differenced on the child competence scale and the parent competence scale based on parent questionnaireweek 2, 3 and 4 week postpartum

**Child** **Competence**	**Week 2**		**Week 3**		**Week 4**	
	Total population (*n* = 37)		Total population (*n* = 30)		Total population (*n* = 27)	
Mean (SD)	2.97 (0.66)	*p* < .001	2.99 (0.76)	*p* < .001	2.640 (0.88)	*p* < .001 d = 0.70
	Intervention group (*n* = 20)	d = 0.53	Intervention group (= 19)	d = 0.60	Intervention group (*n* = 14)	
Mean (SD)	3.35 (0.47)		3.36 (0.54)		3.18 (0.62)	
	Control group (*n* = 17)		Control group (*n* = 11)		Control group (*n* = 13)	
Mean (SD)	2.54 (0,59)		2.38 (0.70)		2.08 (0,77)	
**Parent** **Competence**	**Week 1**		**Week 2**		**Week 3**	
	Total population (*n* = 37)		Total population (*n* = 30)		Total population (*n* = 27)	
Mean (SD)	2.95 (0.68)	*p* < .001	2.97 (0.74)	*p* < .001 d = 0.62	2.81 (0.89)	*p* < .001
	Intervention group (*n* = 20)	d = 0.53	Intervention group (= 23)		Intervention group (*n* = 17)	d = 0.74
Mean (SD)	3.26 (0.57)		3.29 (0.55)		3.30 (0.69)	
	Control group (*n* = 19)		Control group (= 13)		Control group (*n* = 14)	
Mean (SD)	2.57 (0,62)		2,41 (0,72)		2,29 (0,80)	

d = Cohen’s d

##### The emotional availability scales (T5)

The video-recordings of mother-child interaction were coded using the Emotional Availability Scales (EA Scales) 4th edition [27]. The EA Scales assess multiple components of the caregiver-child relationship from the perspective of both partners. The four caregiver scales measure sensitivity, structuring, non-intrusiveness, and non-hostility; two scales measure the child’s responsiveness to the caregiver, and involvement of the caregiver. Each of the six scales is scored on a Likert-type scale. For data analysis purposes, a mean score for each scale is used. Construct validity has been established in longitudinal studies with specific application to interactions between mothers with depression and their children [28]. Test-retest reliability of mothers and their five-month-old infants was observed using intra-class correlations in a two-way, random effects model [29]. EAS has been validated in various settings and cultures by coding a 20-minute filmed interaction [27, 30–32].

The video recordings were coded by certified EAS coders who were blinded to group participation.

### Ethical considerations

The study has been performed in accordance with the Declaration of Helsinki and approved by the National Bioethics Committee of Iceland 21/06/2016 (VSN-16-095/06–16). A prerequisite for participation in the study was informed and written consent from the medical and nursing directors of Primary Health Care in the Capital area. The trial is registered in ClinicalTrials.gov with the identifier: 02/01/2021 (NCT04739332N).

### Analysis

#### Statistical analyses

Baseline differences between groups were merely examined based on descriptive information. Logistic regression analyses with group assignment as an independent variable were carried out at each measurement time point to assess drop-out after baseline. Two sample t-tests were also carried out to compare parent summary forms between groups at the three time points. Mixed effects models for child and parent competence were used to estimate differences between the intervention (G1) and control (G2) groups at the three time points. Two sample t-tests were used to compare intervention and control groups’ EAS scores at 4 months. These comparisons were repeated using linear regression with adjustment for child gender, breastfeeding (full, partial, none) and gestation length. First, a model with adjustment for time only was estimated. Data were analysed using SPSS version 27 [33] and the R package nlme [34].

## Results

### Study population

The study sample comprised 54 mothers and infants, 26 mothers and infants in the intervention group and 28 mothers and infants in the control group.

The characteristics of the study population are shown in Table 4.

**Table 4:** Participant characteristics

	Total population	Intervention group (G1)	Control group (G2)
**Mother**			
EPDS Score ≥ 10 n (%)	12 (22.6)	5 (18.5)	7 (29.6)
History of depression n (%), *n* = 51 (25 + 26)^a^	26 (51.0)	14 (56.0)	12 (46.2)
History of Anxiety n (%), *n* = 51 (25 + 26)^a^	44 (86.3)	20 (80.0)	24 (92.3)
Maternal age mean (SD)	29.7(5.7)	27.9 (5.2)	31.3 (5.8)
Children, *n* = 47 (25 + 22)^a^			
First-time mother n (%)	32 (68.1)	16 (64.0)	16 (72.7)
1 older child n (%)	8 (17.0)	5 (20.0)	3 (13.6)
2 older children n (%)	6 (12.8)	4 (16.0)	2 (9.1)
Maternal education, *n* = 48 (25 + 23)^a^			
Primary school n (%)	4 (8.3)	4 (16.0)	0 (0.0)
Apprenticeship n (%)	3 (6.3)	2 (8.0)	1 (4.3)
Graduated n (%)	11 (22.9)	4 (16.0)	7 (30.4)
University/college n (%)	15 (31.3)	7 (28.0)	8 (38.4)
Master/PhD n (%)	9 (18.8)	4 (16.0)	5 (21.7)
Other	6 (12.5)	4 (16.0)	2 (8.7)
Work position,, *n* = 48 (25 + 23)^a^			
80–100%	30 (62.5)	13 (52.0)	17 (73.0)
< 50%	1 (2.1)	0 (0.0)	1 (4.3)
Financial status, *n* = 48 (25 + 23)^a^			
Very good/Good	22 (45.8)	11(44.0)	11 (47.8)
**Child**			
Male n (%), *n* = 37 (20 + 17) ^a^	23 (62.2)	14 (70.0)	9 (52.9)
Gestation age n (%), *n* = 37 (20 + 17) ^a^			
< 37 weeks	1 (2.7)	1 (5.0)	0 (0.0)
Birth weight in grams n (%)			
*n* = 37 (20 + 17) ^a^ < 2800	2 (5.4)	1 (5.0)	1 (5.9)
2800–3500^−^	13 (35.1)	6 (30.0)	7 (41.2)
3500–4000	18 (48.6)	12 (60.0)	6 (35.3)
>4000	4 (10.8)	1 (5.0)	3 (17.6)

^a^ Intervention and control group

### Maternal confidence

The mothers’ evaluations of the three home visits in weeks 2, 3 and 4 postpartum based on the Parent questionnaire are shown in Table 5. The table illustrates group mean scores and SD for the 7 (+ 1) items from the Parent questionnaire that was completed after the home visit had taken place in weeks 2, 3 and 4 postpartum.

**Table 5:** Group mean scores and SD for the parent questionnaire in weeks 2, 3 and 4 postpartum

Child Competence: How much did you learn about your baby’s competencies:	Mean (SD) Week1		Mean (SD) Week2				Mean (SD) Week3			
	G1	G2			G1	G2	G1	G2		
What he is able to do now	3.50 (0.51)	2.94(0.83)	3.26 (0.65)			2.82 (0.75)	3.21(0.58)	2.54(1.05)		
How your baby can communicate through his/her behaviour	3.70(0.47)	2.59(0.71)	3.68(0.48)			2.64(0.92)	3.43(0.65)	2.15(0.80)		
How your baby can protect his/her sleep	3.15(0.93)	2.29(0.92)	3.16(0.77)			2.00(0.94)	3.00(0.96)	1.69(0.86)		
Did the home visit help you get to know your baby more	3.05 (0.83)	2.35(0.70)	3.32(0.82)			2.09(0.83)	3.07(0.99)	1.92(0.76)		
**Parent Competence**: **How much did you learn about**										
How you can respond to his/her behaviour	3.30(0.57)	2.59(0.79)	3.32(0.58)			2.45(0.82)	3.43(0.65)	2.23(0.93)		
How you can help your baby when he/she is crying	3.40(0.60)	2.65(0.86)	3.32(0.58)			2.27(0.93)	3.43(0.65)	2.38(0.96)		
How to interact with him/her	3.45(0.69)	2.59(0.71)	3.32(0.58)			2.27(0.91)	3.36(0.63)	2.15(0.99)		
Feel more confident as a parent	2.90 (1.02)	2.47(0.87)	3.31(0.92)			2.45(0.82)	3.00(1.18)	2.38(0.77)		

*1 = nothing, 2 = a little, 3 = some, 4 = a lot

*G1 = Intervention group, G2 = Control group

Table 3 shows group differences on the ‘Child competence scale’ and ‘Parent competence scale’ developed from the Parent questionnaire. The results show that, compared to the mothers in G2, the mothers in G1 reported significantly higher learning outcomes on both scales (Child competence and Parent competence) after all three NBO sessions.

In Table 6, the models show a higher score in the NBO group, for both child and parent competence.

**Table 6:** Models adjusted for time only

Child competence	Estimate	CI	*P* value
Intervention vs. control	1.01	0.71, 1.31	< 0.001
Time			0.030
NBO 2 vs. NBO 1	-0.06	-0.27, 0.15	0.574
NBO 3 vs. NBO 1	-0.29	-0.52, -0.07	0.010
Parent competence			
Intervention vs. control	0.90	0.57, 1.23	< 0.001
Time			0.802
NBO 2 vs. NBO 1	0.02	-0.19, 0.22	0.878
NBO 3 vs. NBO 1	-0.06	-0.27, 0.16	0.606

### Intervention effects on mother-infant interaction

Two-sample t-tests yielded no evidence of differences in EAS scores on the 6 scales (4 parent scales and 2 child scales) between the intervention and control groups at T4 (Table 7, *p* ≥ .383). In adjusted analyses, however, there was some evidence of a higher score on the non-hostility scale in the intervention group (*p* = .031), but not for the other EAS scales (*p* ≥ .118).

**Table 7:** EAS scale scores at 4 months, with two sample t-tests

	Descriptive		Unadjusted			Adjusted		
EAS scale	Interv.	Control	Diff	CI	p	Diff	CI	p
Sensitivity	23.87 (29)	23.76 (29)	0.11	-2.18, 2.39	0.926	1.55	-1.56, 4.67	0.319
Structuring	24.02 (26)	23.60 (29)	0.42	-2.07, 2.90	0.738	2.58	-0.69, 5.86	0.118
Non-intrusiveness	23.72 (25)	23.19 (29)	0.53	-2.20, 3.26	0.701	1.82	-1.50, 5.14	0.273
Non-hostility	26.60 (26)	25.93 (29)	0.67	-0.85, 2.18	0.383	1.90	0.19, 3.62	0.031
Responsiveness	24.08 (26)	23.71 (29)	0.37	-1.67, 2.41	0.718	1.37	-1.36, 4.10	0.314
Involvement	21.94 (26)	21.12 (29)	0.82	-1.30, 2.94	0.441	1.98	-0.97, 4.94	0.182

Descriptive information, mean (n). Diff, difference between Intervention-Control. p, p-value. CI, 95% confidence interval. Unadjusted, two-sample t-test. Adjusted, linear regression adjusted for child gender, breastfeeding and gestation length. Interv., intervention

## Discussion

This randomised controlled trial is the first study carried out in Iceland to investigate effects of the NBO intervention on post-partum mothers with some risk factors associated with disturbed mother-infant interaction and negative child outcomes. Our first hypothesis, which stated that mothers in G1 will show higher sensitivity when interacting with their infant compared to the mothers in G2 at 4 months postpartum, and that the infants in G1 will show better regulatory capacity than the infants in G2, was mainly not confirmed. The only significant group difference in mother-infant interaction was lower hostility among the mothers in the intervention group. This difference was, however, only significant in adjusted analyses. Our second hypothesis, which stated that the mothers in the intervention-group (G1, NBO combined with standard care) will experience higher satisfaction and benefits from the postpartum home visits compared to the mothers in the control group (G2, standard care only), was confirmed.

The results for the Parent questionnaire completed after each home visit in weeks 2, 3 and 4 postpartum showed that the mothers in the intervention group scored significantly higher on both the Parent competence scale and the Child competence scale compared to the mothers in the control group. The intervention mothers reported that the public health nurse helped them to know their infants better, that they learned about their infant’s abilities, how the infants communicated through nonverbal behaviour and how their infant can protect his/her sleep. Furthermore, they learned how to respond to the infant’s behaviour, how they could help when he/she was crying, how to interact with the infant. In general, they felt more confident as a parent after the three home visits by a NBO-certified public health nurse.

Our findings of increased maternal confidence are consistent with several previous studies on NBO [18–20, 22, 35, 36]. Two of these studies were conducted in other Nordic countries, Norway, and Denmark, respectively. In these studies, the NBO was tested as a universal preventive intervention delivered by health practitioners in child healthcare centres. Both studies found that the intervention group learned significantly more about the infant’s signals and needs than the group that received standard follow-up care from the child healthcare centres. A similar positive effect on maternal confidence was reported in an Australian study of at-risk mothers only [18]. One key impact of the NBO sessions is that they allow the parent to experience moments in which the newborn infant shows his innate competence and learn how to respond and interact sensitively with the baby. These findings thus correspond well with the content and the aim of the intervention [17], indicating that the NBO intervention provides parents with a better grasp of the baby’s signals and needs in important everyday situations.

Since healthy parent-child interactions depend on the caregiver’s capacity to comprehend and be attuned to the infant’s internal state and behaviour [5], our findings are important and may have clinical implications for postpartum follow-up.

Our study produced no robust results concerning increased maternal sensitivity or child regulatory capacity in the intervention group measured by EAS when the infant was 4 months of age. However, in adjusted analyses, there was some evidence of a higher score on the EAS non-hostility scale in the intervention group (*p* = .031), but not on the other EAS scales (*p* ≥ .118). The group difference for non-hostility suggests that the mothers in the intervention group showed fewer signs of negative attitudes and interpretations of the infant’s behaviour during their interaction. The three NBO sessions in the first month postpartum may have increased the mother’s understanding of the newborn infant’s strong need for other-regulation and led to increased empathy for the infant when dysregulated or unfocused. The mothers may have strengthened their ability to understand the infant from the inside, not only from the outside.

Some indication of heightened sensitivity in mother-infant interaction was also reported in the Australian NBO study of an at-risk sample. More specifically, the researchers reported higher EAS sensitivity and non-intrusiveness scores with medium effect size in the intervention group [18]. The Australian study and our study have many similarities, such as three NBO sessions after birth and use of the EAS to assess maternal sensitivity at 4 months postpartum. Together, our studies provide some evidence of the effectiveness of NBO in terms of improved parent-infant interaction as well as maternal confidence. There are several factors that may have contributed to the weak confirmation of our first hypothesis. Intervention effects may not be strong enough to be visible in behaviour (NBO dosage may not have been enough), and we would need a larger sample size to find small to moderate effects. It may also be that stronger effects would appear at a later stage than four months postpartum. Another factor worth noting is the universal comprehensive follow-up programme for postpartum women and their infants in Iceland. Every family receives 13 consultations in the first 3 months, which certainly makes it difficult to observe significant differences in the mother-infant relationship at 4 months postpartum.

### Strengths and limitations

Like all studies, our intervention study has strengths and limitations. We used a robust design and measures based on self-reports and observation data that make it possible to compare findings with other NBO studies. The EAS coders were blinded to group belonging. Moreover, the study was conducted in a real-world clinical setting, and it provides valuable data on the effects of NBO intervention in at-risk populations. The small sample size and lack of a fidelity measures are obvious weaknesses and the power to detect differences may have been affected the results. The parent questionnaire results should be interpreted with caution, particularly regarding the subscales, due to the lack of formal validation. Hence, the results must be interpreted with some caution.

## Conclusion

The results of this randomised study appear to support the idea that health practitioners can use the NBO to strengthen maternal confidence during the first month with their newborn baby and it may also have a potential to lead to lower hostility among the mothers. Further research using a bigger sample size and longer follow-up is highly warranted.

## Data Availability

The data that support the findings in this study are available by contacting SBA, but restrictions apply to the availability of these data, which were used under licence for the current study, and are thus not publicly available. Data are however available from the author upon reasonable request and with the permission of the Icelandic National Bioethics Committee.

## Abbreviations

NBO: Newborn Behavioural Observations System
